# Binding properties and biological applications of green synthesized ZnO nanoparticles from neem flower

**DOI:** 10.1038/s41598-025-02157-x

**Published:** 2025-05-22

**Authors:** Palanivelmurugan Mohanasundaram, Mary Saral A.

**Affiliations:** Department of Chemistry, School of Advanced Sciences, Vellore Institute of Technology, Vellore, Tamil Nadu 632014 India

**Keywords:** Binding property, Cytotoxicity, Green synthesis, Neem flower, Zinc oxide NP, Chemical biology, Plant sciences, Chemistry, Materials science, Nanoscience and technology

## Abstract

Zinc oxide (ZnO) nanoparticles exhibit distinctive electronics, sensors, and biomedical applications. The present work aims to synthesize an eco-friendly, cost-effective, and sustainable approach for the fabrication of zinc oxide nanoparticles (ZnO NPs). An aqueous extract of neem flowers was used to fabricate the ZnO NPs as a natural reducing and capping agent. Analytical techniques, including UV-Vis, TEM, FTIR, FESEM, XRD, zeta potential analysis, and TGA confirmed the successful formation, crystalline nature, and stability of the ZnO NPs. The synthesized ZnO NPs were confirmed by the peak at 363 nm in the UV-Vis spectrum. The average particle size was found between 30 ~ 60 nm by FESEM, TEM, and Particle Size Analysis. The ZnO NP shows the highest % of inhibition was found to be > 80% in antioxidant activity. The antimicrobial assays show promising activity against pathogens. Moreover, their interaction with biomolecules such as DNA and bovine serum albumin (BSA) demonstrated strong binding affinity, highlighting their potential for biomedical applications. Cytotoxicity analysis using HEK-293 cells revealed minimal toxicity, suggesting that these nanoparticles are safe for normal cells. This study highlights the potential of green-synthesized ZnO nanoparticles as a promising option for therapeutic, antimicrobial, and bioengineering applications.

**Supplementary Information:**

The online version contains supplementary material available at 10.1038/s41598-025-02157-x.

## Introduction

Metal oxide nanoparticles are gaining significant attention for their innovative applications in medicine, biology, sports equipment, electronics, solar energy, magnetics, and various other industries^1,2^. Its cost-effectiveness, minimal toxicity at low concentrations, chemical stability, and customizable optical and electrical properties make it highly adaptable for use in environmental science, chemistry, physics, biology, medicine, and electronics^3–5^. ZnO nanoparticles are notable among metallic nanoparticles for their biocompatibility, potential antibacterial properties, and favorable optical, electrical, and photocatalytic characteristics^6,7^. The properties are primarily influenced by factors such as size, shape, composition, morphology, crystalline phase, and surface area^8^. The multifaceted benefits of nanoparticles have increasingly been harnessed for real-time applications, bridging the gap between laboratory research and practical implementation^9,10^. The modern human population faces numerous challenges, such as cancer, bacterial and fungal infections. Cancer is a multifaceted disease characterized by the unchecked growth and spread of abnormal cells. It remains one of the leading causes of death globally, affecting millions of people annually^11^ Common treatments, including surgery, chemotherapy, and radiation, often involve high costs, and lengthy procedures, which are accompanied by side effects^12^. Similarly, microbial infections, resulting from harmful microorganisms like bacteria, viruses, and fungi, can cause diseases ranging in severity from mild to serious. If left untreated, they can become life-threatening^13–15^. To overcome these, suitable, potential, eco-friendly, and sustainable alternatives are required. Metal oxide nanoparticles possess unique properties which makes them highly valuable in various biomedical applications. These characteristics are particularly important in the fight against complex health challenges, including infections and cancer^16^. As traditional treatments often face limitations like drug resistance and side effects, the multifunctional nature of metal oxide nanoparticles offers promising alternatives for targeted therapy, drug delivery, and antimicrobial treatments^17^. Zinc oxide (ZnO) NPs are widely examined for their anticancer, antibacterial, and antifungal properties for their biocompatibility and selective targeting ability of cancer cells. Numerous studies have explored synthesizing ZnO nanoparticles using plant extracts for anticancer applications^18,19^. Green synthesis, which utilizes plant-based methods, is preferred over conventional approaches involving hazardous chemicals^20^. In this eco-friendly process, plant-derived phytochemicals serve as reducing and capping agents. The compounds introduce functional groups like alcohols, carbonyls, and amines, enhancing nanoparticle targeting efficiency^21^. Green synthesis is also cost-effective, fast, and safe, allowing for better control over nanoparticle size, surface area, and morphology^22,23^. Numerous studies have extensively reported the plant-mediated synthesis of ZnO nanoparticles, highlighting their remarkable biological activities^24,25^. In this study, we present a plant-mediated synthesis approach for ZnO NPs using aq. *Azadirachta indica* (neem) flower extract as a reducing agent. The synthesized NPs were characterized through UV-Vis, FTIR, FESEM, TEM, DLS & zeta potential measurements. Furthermore, ZnO NPs were evaluated for their pharmaceutical activities including their antioxidant, antibacterial, and antifungal properties, DNA and bovine serum albumin (BSA) binding interactions, and in-vitro cytotoxicity potential.

## Materials and methods

### Reagents and solvents

All the metal salts and solvents used in this study were procured from Merck, India, and were of analytical grade, ensuring high purity and reliability for experimental procedures. Throughout the entire experiment, double-distilled water was employed at every stage to maintain consistency, eliminate potential contaminants, and enhance the accuracy of the results. This rigorous approach helped ensure the precision and reproducibility of the experimental findings.

### Preparation of neem flower extract and phytochemical analysis

From a single neem tree, flowers were harvested in Vellore during February and March 2020. After collection, the flowers were shade-dried and stored in an airtight container for subsequent use. The extraction process and phytochemical analysis of the neem flower extract were conducted following the methods outlined in our previous studies^26^.

### Synthesis of ZnO nanoparticles

Zinc oxide nanoparticles (ZnO NPs) were prepared using neem flower extract in water, following a slightly adjusted version of a known method^27^. To begin, 10 mg of concentrated neem flower extract was mixed into 20 mL of water. This mixture was then slowly added drop by drop to a zinc sulfate solution (1.5 g ZnSO₄ dissolved in 100 mL of water) while stirring with a magnetic stirrer at 80 °C. The solution was stirred until it turned a yellow-orange color, then left at room temperature for two hours. After that, the mixture was spun in a centrifuge at 7000 rpm for 15 min. The solid formed was washed several times with water and ethanol to remove any leftover impurities. The cleaned material was then dried at 100 °C for two hours, ground into a powder, and heated at 500 °C for another two hours. Finally, the prepared powder was stored in a sealed container at 4 °C for later use and testing.

### Characterisation

The synthesized ZnO NPs were characterized using a range of analytical tools to confirm the nanostructure (see SI file, page 3).

###  Antioxidant activity

The antioxidant potential of ZnO NPs were evaluated using different methods^27–29^.

#### DPPH radical scavenging activity

The antioxidant capacity of ZnO nanoparticles (NPs) was evaluated using a DPPH assay. Different concentrations of ZnO NPs (20–100 µg/mL) prepared in methanol were combined with a 1 mM DPPH solution and allowed to incubate at rt for 30 min. The abs of the samples and the control was then recorded at 517 nm. The percentage of inhibition was calculated as follows:

$$\% {\text{ inhibition }}={\text{ }}\left[ {\left( {{\text{Ab}}{{\text{s}}_{{\text{control}}}} - {\text{ Ab}}{{\text{s}}_{{\text{sample}}}}} \right){\text{/Ab}}{{\text{s}}_{{\text{control}}}}} \right] \times {\text{1}}00.$$

#### Reducing power assay

ZnO nanoparticles (NPs) were tested at concentrations ranging from 20 µg/mL to 100 µg/mL. The samples were combined with 0.2 M phosphate buffer and 1% potassium ferricyanide, with ascorbic acid serving as a positive control. The reducing power was evaluated by measuring the absorbance of the samples at 700 nm.

#### Phospho-molybdenum assay

ZnO nanoparticles (NPs) were tested at concentrations ranging from 20 to 100 µg/mL. Each test tube contained the ZnO NPs, 3 mL of dd water, and 1 mL of molybdate reagent. The samples and ascorbic acid abs. was measured at 695 nm.

### Antimicrobial activity

The antibacterial activity of ZnO nanoparticles (NPs) was evaluated using the traditional disc diffusion method^30,31^. The antimicrobial activity of the extracts was assessed following a reported protocol, with dimethyl sulfoxide (DMSO) serving as a negative control. Different concentrations of ZnO NPs (25, 50, 75, and 100 µg/mL) were tested against *Staphylococcus aureus (ATCC 25923)*,* Pseudomonas aeruginosa (ATCC 27853)*,* Candida tropicalis (ATCC 10231) and Candida glabrata (MTCC 3019)* Standard antibiotics (ampicillin for bacteria and ketoconazole for fungi) were used as positive controls. All experiments were performed in triplicate, with the plates incubated at 37 °C for 24 h. The inhibition zones surrounding each disc were measured and documented in millimeters (mm).

### DNA binding study

The DNA binding ability of the extracts was investigated following established protocols^32,33^. The sample was prepared in a Tris-HCl/NaCl buffer, and the concentration of calf thymus DNA (CT-DNA) was determined from its absorbance spectrum. Titration was performed by gradually increasing the CT-DNA concentration with absorbance measurements taken after each addition. A plot of [DNA]/(εa − εf) vs. [DNA] was constructed, and the binding constant (K_b_) was determined from the linear fit of the plot using the equation:

1 $$\:\frac{\left[DNA\right]}{{\epsilon\:}_{a}-{\epsilon\:}_{f}}=\frac{\left[DNA\right]}{{\epsilon\:}_{b}-{\epsilon\:}_{f}}+\frac{1}{{K}_{b}({\epsilon\:}_{a}-{\epsilon\:}_{f})}$$

### BSA binding study

Serum albumin proteins play a crucial role in drug transport and metabolism within the bloodstream. The structural similarity between human serum albumin (HSA) and BSA makes BSA a useful model for quenching studies. In binding studies, BSA was used to assess the drug-protein binding capacity^32,33^. Tris-HCl/NaCl buffer was used to prepare A BSA solution (2 × 10^6^ M) was prepared BSA solution (2 × 10^6^ M) and the aqueous samples were added to this solution. The fluorescence intensity of BSA was measured, and a decrease in fluorescence indicated the interaction between BSA and the compounds. The quenching constant (K_BSA_) was calculated using the Stern-Volmer equation. Additionally, the number of binding sites (n) was determined using the Scatchard equation (equation vi).

2 $$\:{I}_{0}/I=1+{K}_{BSA}\left[Q\right]=1+{k}_{q}{\tau\:}_{0}\left[Q\right]$$

3 $$\:{\text{l}\text{o}\text{g}(I}_{0}-I/I)=\text{log}K+n\text{log}\left[Q\right]$$

### Cytotoxicity

The cytotoxic potential of synthesized nanoparticles has been evaluated using established protocols (see SI, page 3)^34^. The percentage of cell growth inhibition and the IC_50_ value were calculated from the dose-response curves.

$$\% {\text{ Inhibition}}\,=\,{\text{1}}00{\text{ }}--{\text{ }}\left( {{\text{OD of sample}}/{\text{OD of Control}}} \right){\text{ }} \times {\text{ 1}}00.$$

## Results and discussion

### Phytochemical analysis

Plant-derived compounds exhibit significant potential as natural reductants or oxidants due to their biologically active constituents, making them suitable candidates for the synthesis of metallic nanoparticles^18–20^. In this study, phytochemical screening was performed on the aqueous flower extract of *Azadirachta indica A. Juss*, employing standard qualitative procedures^26^. The presence of alkaloids, flavonoids, phenolics, carbohydrates, and saponins was confirmed. These bioactive compounds are likely the key contributors to the reduction and stabilization processes involved in nanoparticle formation. The results align with prior studies reporting the presence of a diverse array of bioactive compounds in *Azadirachta indica* extracts^26^. This study highlights the potential application of *Azadirachta indica* in green nanotechnology, utilizing its phytochemicals for environmentally sustainable nanoparticle synthesis.

**Fig. 1:**
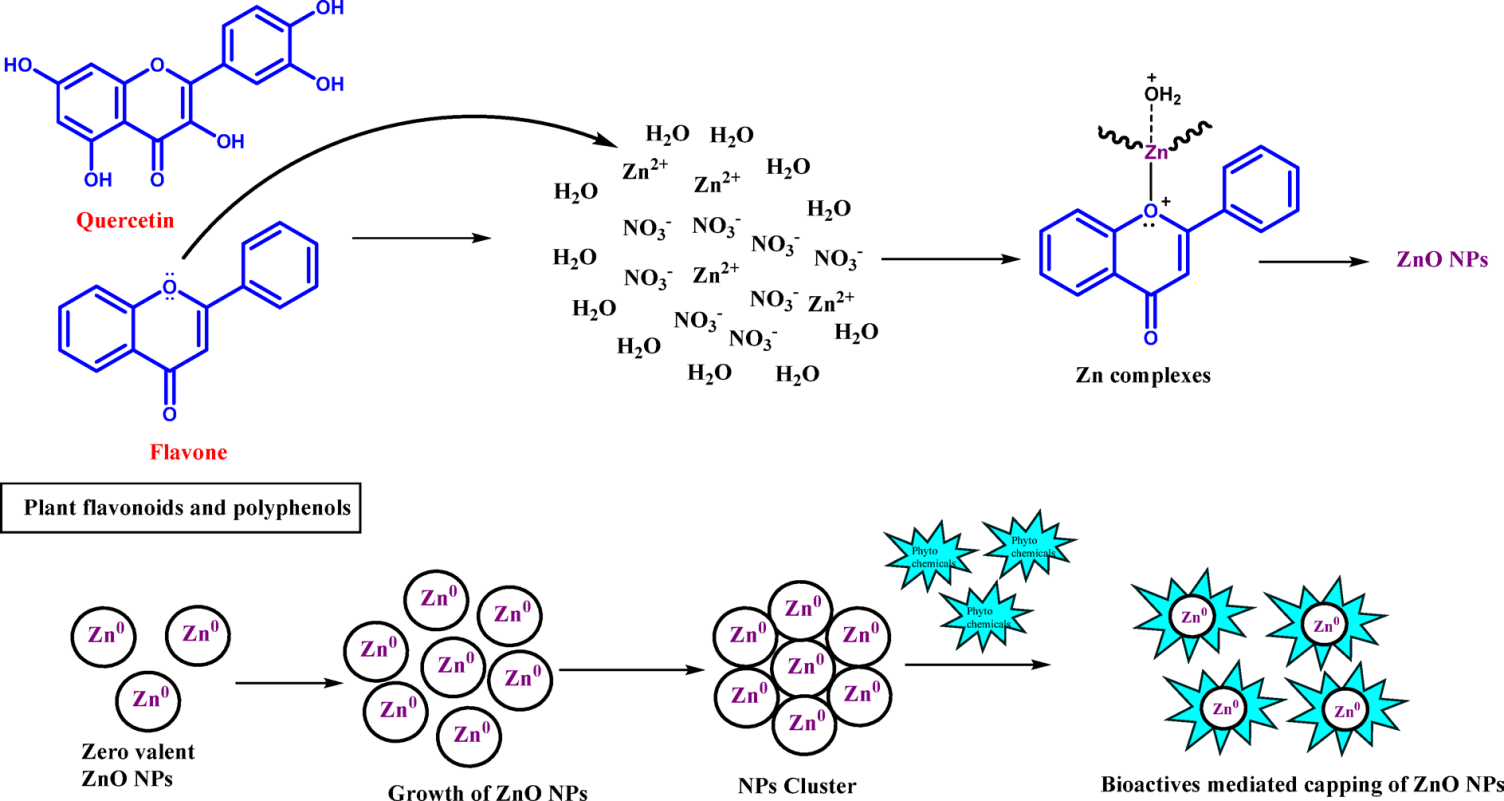
Plausible mechanism of formation of ZnO NPs by phytochemicals.

The plausible mechanism of the formation of ZnO NPs using phytochemicals present in the neem flower extract which act as a reducing, capping, and stabilizing agent is represented in Fig. 1. The figure represents the formation of ZnO nanoparticles by phytochemicals (i.e., flavones, quercetin, etc.). Phytochemicals play a pivotal role in the formation of ZnO NPs. It donates electrons to Zn^2+^ ions and reduces them to zerovalent Zn^0^ atoms. It also participates in the growth step to stabilize and reduce irregularities in the morphology and regulate the size of the nanoparticles (Fig. 1).

### Characterisation of G-ZnO NPs

####  Analysis of UV-Vis spectrum and band gap energy calculations

**Fig. 2:**
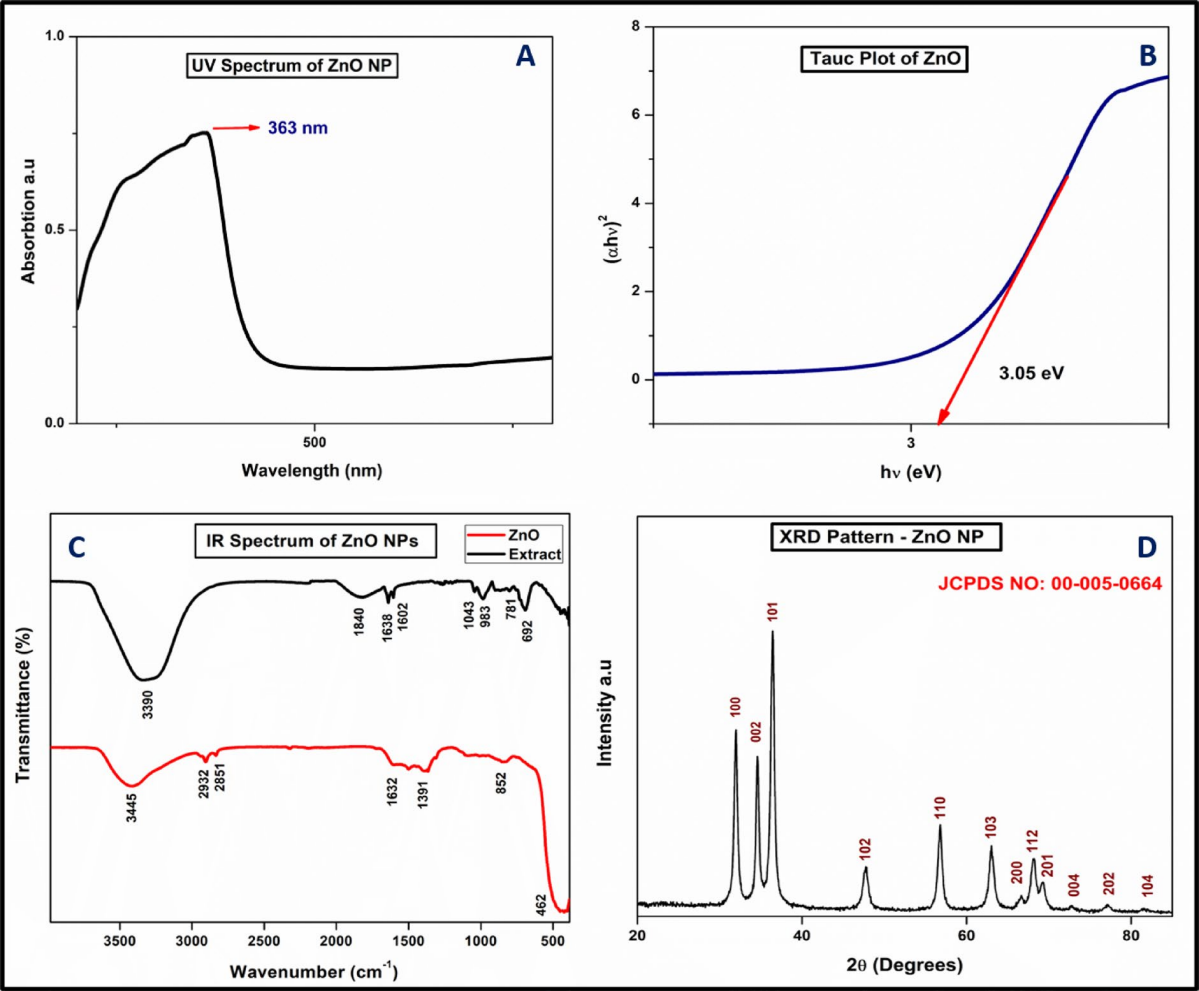
(**A**) UV-Vis spectrum of ZnO NPs, (**B**) Tauc Plot of ZnO, (**C**) IR spectrum of ZnO NPs & Flower Extract (**D**) pXRD pattern of ZnO.

The UV-Vis absorption spectrum of the biosynthesized ZnO nanoparticles (ZnO NPs) is shown in Fig. 2A, revealing a distinct absorption peak at 363 nm. This observation aligns with previously reported studies^26^. The excitonic absorption of the biogenically synthesized ZnO NPs exhibits a notable blue shift at 363 nm, reflecting size-dependent optical properties. The sharp and well-defined absorption peak indicates the formation of monodispersed nanoparticles, likely due to surface plasmon resonance. This blue shift carries significant implications regarding the nanoparticle size and electronic properties. The observed blue shift indicates that the nanoparticles are relatively small. As particle size decreases, especially below the Bohr exciton radius, the energy levels become discretized, and electron-hole pairs (excitons) require more excitation energy. This leads to the absorption of shorter wavelengths (higher energy), thus causing a blue shift in the spectrum. In metallic nanoparticles (e.g., Ag, Au), a blue shift in the surface plasmon resonance (SPR) peak typically suggests a decrease in particle size. In semiconductor nanoparticles (e.g., ZnO, CdS), it reflects quantum size effects altering the band gap. Therefore, the shift to 363 nm suggests a successful synthesis of ultrasmall nanoparticles with modified optical properties. The band-gap energy of the ZnO NPs was calculated as depicted in Fig. 2B. The band-gap energy was determined by extrapolating the linear portion of the Tauc plot, which represents the variation of (αhv)² as a function of photon energy (hv). The band-gap energy values were derived using the Tauc equation^26^.

$${{\upalpha}}{({\text{h}}\upsilon )^{\text{2}}}={\text{ A}}({\text{h}}\upsilon \, - \,{\text{Eg}})$$

In this equation, α denotes the absorption coefficient, h represents Planck’s constant, vv signifies the frequency of the incident light, E_g_ corresponds to the band-gap energy, and A is a proportionality constant. The biosynthesized ZnO nanoparticles exhibit a band-gap energy of 3.05 eV, highlighting their potential for use in semiconductors, optics, and biomedical applications. The high band-gap energy of the ZnO NPs contributes to their excellent catalytic activity, UV filtering properties, wound healing capabilities, and anti-inflammatory effects^35^.

####  Analysis of FTIR and p-XRD

The FTIR spectrum of aqueous extract of neem flower and green-synthesized ZnO nanoparticles (ZnO NPs) is presented in Fig. 2C. The prominent peak at 3445 cm⁻¹ corresponds to O–H stretching vibrations, signifying the presence of hydroxyl groups associated with phenolics and flavonoids in the neem flower extract. The peaks at 2932 cm⁻¹ and 2851 cm⁻¹ represent C–H stretching vibrations, characteristic of aliphatic groups found in phytochemicals. The band observed at 1632 cm⁻¹ corresponds to C = O stretching vibrations, indicating the presence of aldehyde or ketone groups in the extract. A peak at 1391 cm⁻¹ corresponds to C–H bending vibrations, further confirming the involvement of organic components. The absorption at 852 cm⁻¹ is attributed to aromatic stretching vibrations, indicative of aromatic compounds derived from the neem flower extract. The above-mentioned peaks are present in both the extract and ZnO NP which confirms that the phytochemicals present in the extract play a vital role in the formation of ZnO NPs. Finally, the peak observed at 462 cm⁻¹ is assigned to metal-oxygen (Zn–O) str. vibrations, which confirm the formation of ZnO. These functional groups highlight the role of the plant extract as a reducing and capping agent in the synthesis of ZnO NPs.

To assess the crystallinity and phase purity of the synthesized ZnO nanoparticles (ZnO NPs), X-ray diffraction (p-XRD) analysis was performed, as shown in Fig. 2D. The diffraction peaks exhibit clear broadening, confirming the nanoscale dimensions of the particles. The observed prominent peaks, along with their corresponding Miller indices, verify the crystalline structure of the ZnO NPs. The reflection peaks are consistent with the hexagonal wurtzite phase of ZnO, which aligns perfectly with the standard reference pattern (JCPDS card No. 00-005-0664). The average crystalline size of the nanoparticles was estimated to be approximately 13 nm, calculated using the Debye-Scherrer equation.

####  FESEM and EDX

**Fig. 3:**
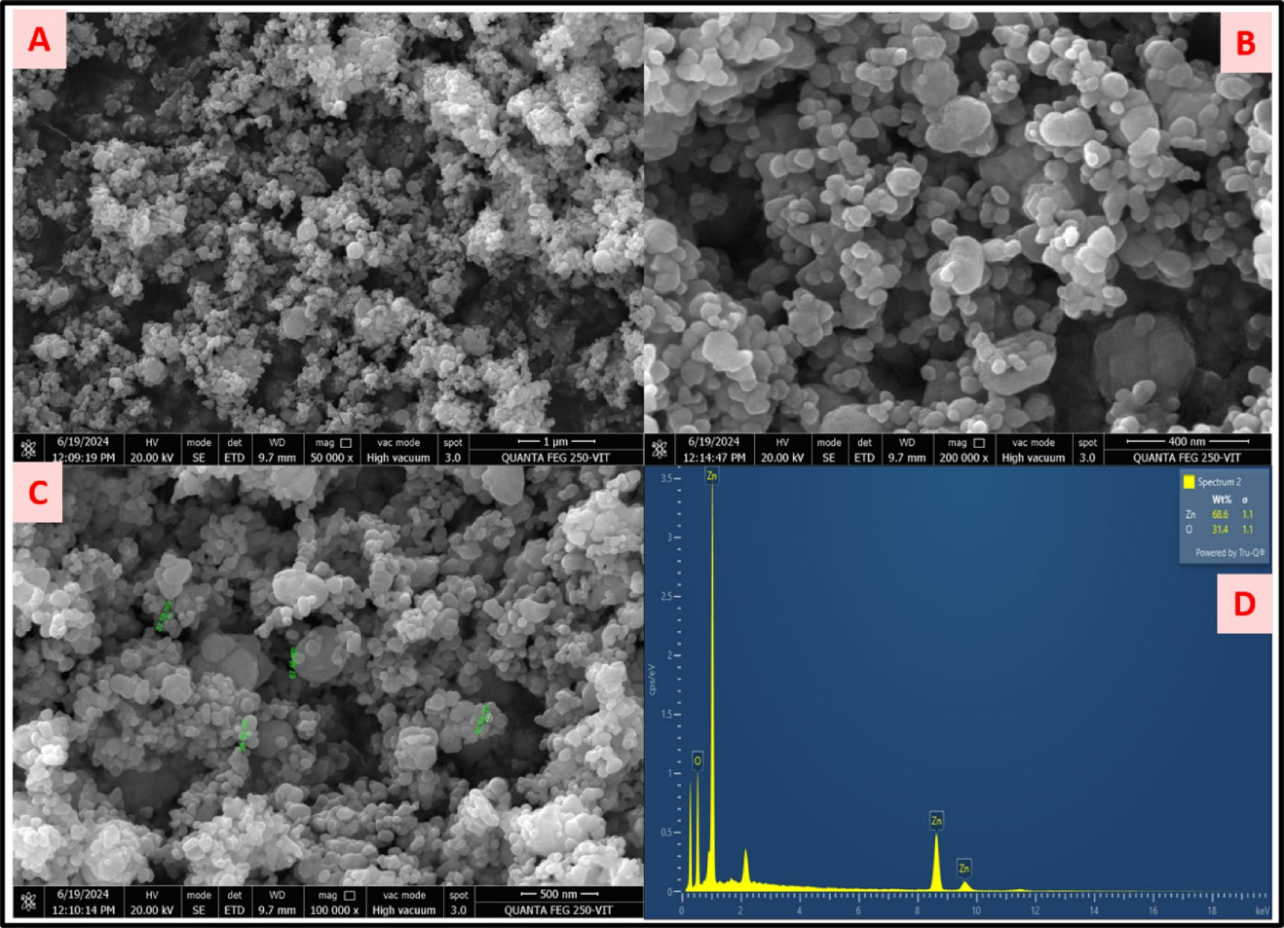
(**A**–**C**) FESEM images of ZnO (**D**) EDAX spectrum of ZnO NPs.

The FESEM images presented in Fig. 3A-C at varying magnifications demonstrate that the synthesized particles exhibit distorted spherical morphologies. This deformation is likely attributed to the presence of pores within the structural framework of the ZnO nanoparticles. Elemental analysis of the nanoparticles was performed using EDX spectroscopy, as shown in Fig. 3D. The EDX spectra revealed prominent peaks corresponding to Zinc and Oxygen, confirming the presence of ZnO. The quantitative analysis yielded elemental compositions of 68.56% Zinc and 31.44% Oxygen, which are consistent with previously reported findings^36^.

The distorted spherical morphologies seen in the FESEM images are due to the irregular nucleation and growth during nanoparticle synthesis. These shape deviations may lead to increased surface area, which can be beneficial for catalytic activity, and biomedical/optical applications. EDX analysis helps clarify whether these morphological anomalies are purely physical or linked to compositional issues. If the elemental composition matches expected values (e.g., the presence of key metal and stabilizing elements), it supports the hypothesis that the core material was successfully synthesized. It also helps to detect impurities or compositional inhomogeneity which indicates incomplete reactions or contamination, challenging the assumed synthesis efficiency. Together, FESEM and EDX results highlight both structural uniformity and compositional purity for reliable nanoparticle performance.

####  TEM, particle size analysis, and zeta potential analysis

**Fig. 4:**
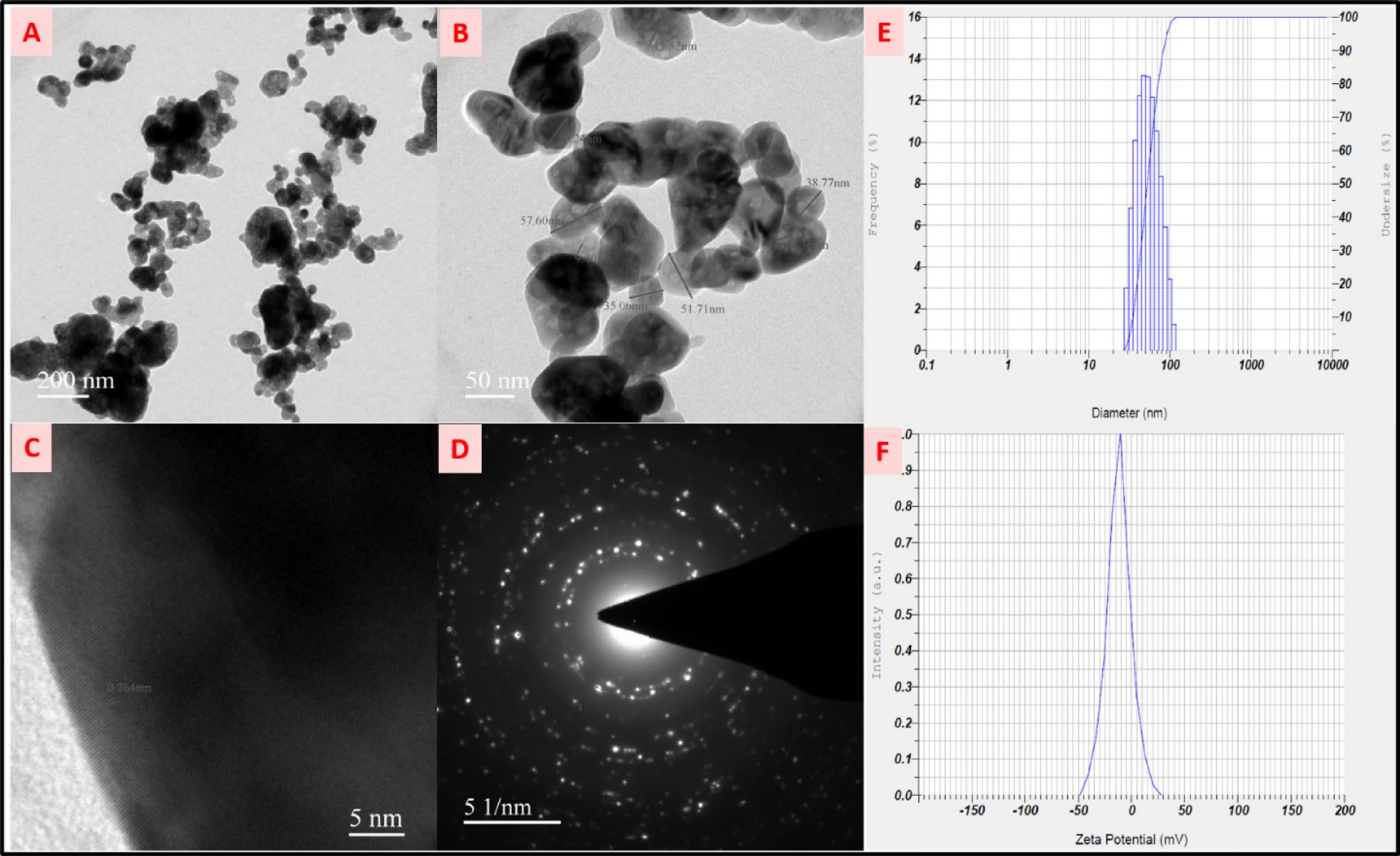
(**A**–**C**) TEM images of ZnO NPs, (**D**) SAED pattern of ZnO NPs, (**E**) particle size analysis of ZnO NPs (**F**) Zeta potential distribution of ZnO NPs.

Transmission electron microscopy (TEM) images of the G-ZnO nanoparticles reveal an average particle size in the range of 30–60 nm (Fig. 4A-C). This size variation may be attributed to the presence of pores within the nanoparticles, which is clearly visible in the high-resolution TEM (HRTEM) images. The average pore size was determined to be approximately 15 nm (Fig. 4B). HRTEM analysis further confirms the crystalline nature of the ZnO NPs, showing well-aligned lattice fringes. The calculated interplanar spacing (d-spacing) is 0.263 nm (Fig. 4C), aligning with the hexagonal wurtzite structure of ZnO. The selected area electron diffraction (SAED) pattern shows concentric rings, confirming the polycrystalline nature of the synthesized nanoparticles (Fig. 4D).

Particle size analysis (DLS) was performed to determine the size distribution of the ZnO nanoparticles (ZnO NPs) in a dispersed medium. The analysis revealed an average particle size of 56 nm (Fig. 4E). The Polydispersity Index (PDI) was determined using dynamic light scattering (DLS) analysis to assess the size heterogeneity of the NPs in the sample. The measured PDI value was 0.107 ± 0.02, indicating high particle uniformity. Reports suggest that a PDI below 0.3 is suitable for drug delivery applications. These results highlight the potential of the synthesized ZnO nanoparticles for various applications. Zeta potential, a key parameter for assessing the stability and functionality of nanoparticles, was measured for the synthesized ZnO NPs. The zeta potential value was determined to be -22 mV (Fig. 4F). This relatively low zeta potential suggests limited electrostatic repulsion between particles, indicating a tendency for agglomeration in the dispersed medium^37^.

####  Thermogravimetric analysis

**Fig. 5:**
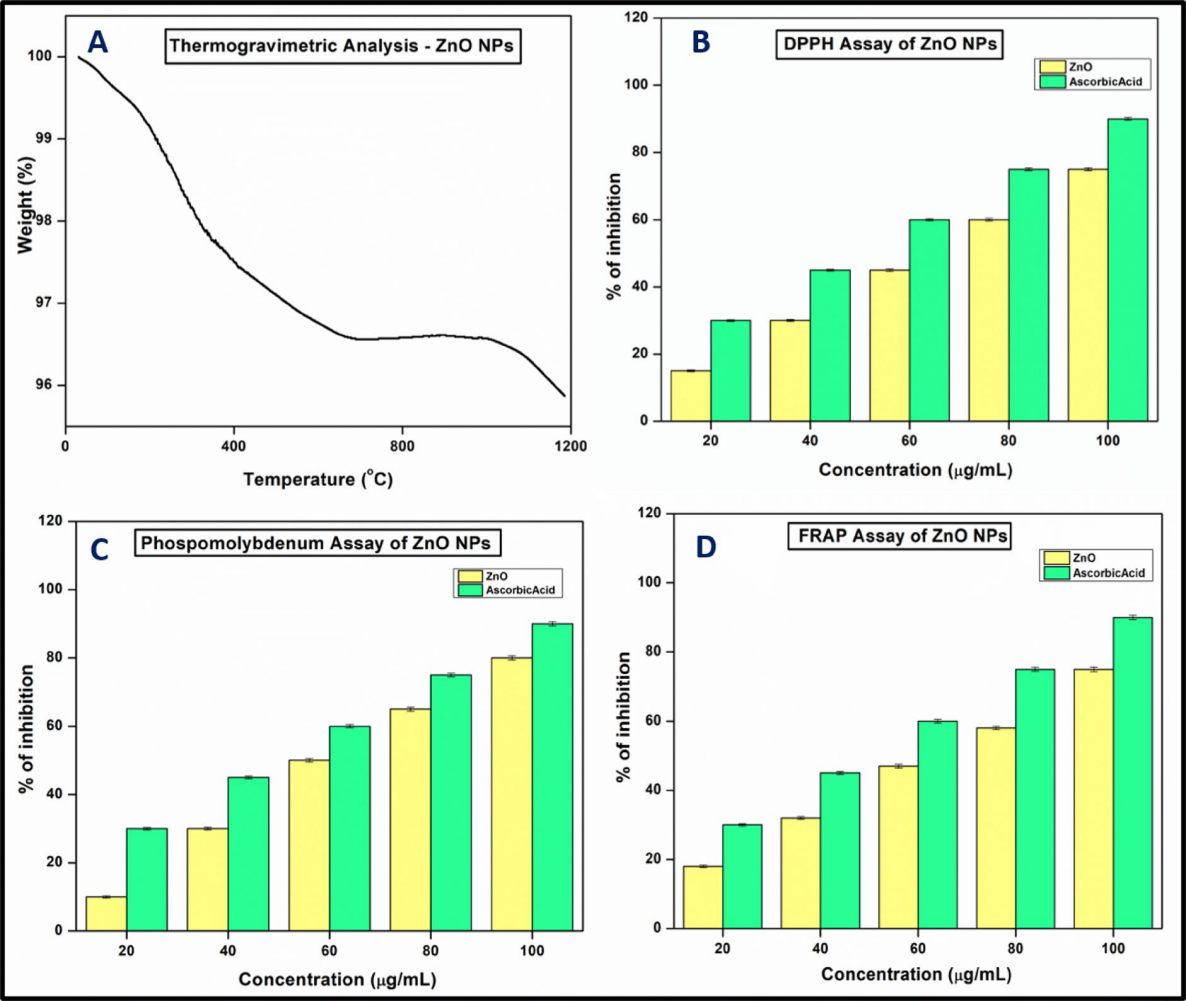
(**A**) Thermogravimetric analysis of ZnO NPs. (**B**–**D**) Antioxidant activity of ZnO of NPs.

The thermal stability of zinc oxide nanoparticles (ZnO NPs) is essential for their use in electronics, optics, and catalysis. TGA analysis shows that the sample undergoes decomposition as the temperature increases, with complete degradation occurring at 1180 °C (Fig. 5A). The first weight loss, around 200 °C, is attributed to the removal of moisture and volatile components that act as a capping agent around the nanoparticles. A second degradation step, near 400 °C, corresponds to the breakdown of organic molecules, such as phenolics, flavonoids, and other biomolecules involved in the biosynthesis of ZnO NPs. The final degradation above 1000 °C may be due to the elemental breakdown of ZnO. The residue left after decomposition was 95.87%, indicating that ZnO NPs maintain excellent thermal stability, with minimal mass loss even at higher temperatures. This stability makes them suitable for a wide range of industrial, technological, and scientific applications^38,39^.

###  Antioxidant activity

The antioxidant activity of ZnO nanoparticles (ZnO NPs) was assessed using three analytical methods: the DPPH assay, ferric-reducing power assay, and the phosphomolybdenum method, with ascorbic acid employed as the standard. The results, depicted in Fig. 5B–D, revealed that all three assays exhibited inhibition rates exceeding 75%, indicating notable antioxidant efficacy. A positive correlation was observed between the concentration of ZnO NPs and the percentage of inhibition, demonstrating a concentration-dependent enhancement of antioxidant activity. The IC_50_ values were determined to be 63.24, 54.11, and 58.25 µg/mL for the DPPH, ferric-reducing power, and phosphomolybdenum assays, respectively. These findings confirm the strong free-radical scavenging capability of ZnO NPs, underscoring their potential as effective natural antioxidants. These results were compared with earlier reports^40,41^. The IC_50_ value of ZnO NPs synthesized using *Punica granutum* was found to be 240 µg/mL^41^ and *Ailanthus altissima* leaf extract-mediated ZnO showed IC_50_ of 78.23 µg/mL^40^ respectively. From our results it is observed that neem flower-mediated ZnO NPs show better antioxidant activity compared to previous reports and found that it has significant importance as a potential antioxidant agent. Also, there is a non-linear correlation between the antioxidant activity and band gap energy of NPs. In molecules like flavanoids, the highest band gap results in the lowest reactivity and vice versa^42,43^. Similarly, ZnO NPs with moderate band gap energy (3.05 eV) have significant antioxidant activity due to their ability to participate in the electron transfer mechanism.

### Antibacterial activity

**Fig. 6:**
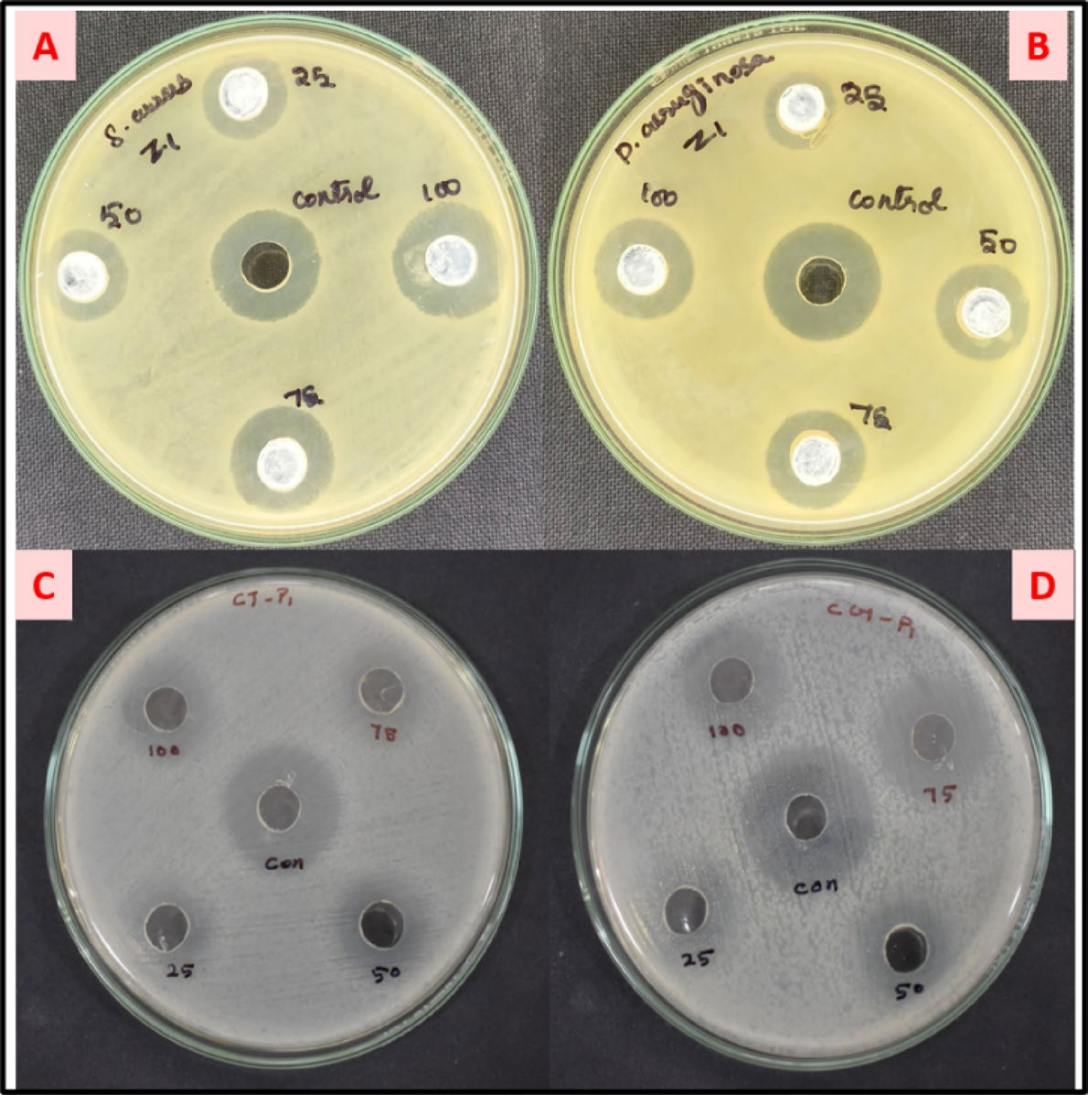
Antibacterial and Antifungal activity of ZnO NPs. (**A**,**B**) antibacterial (**C**,**D**) antifungal.

The antibacterial activity of nanoparticles is crucial for addressing global challenges such as antibiotic resistance, food safety, healthcare-associated infections, and environmental contamination. Their versatility and effectiveness make them a valuable tool for developing innovative solutions across multiple disciplines. Metal oxide nanoparticles (MONPs) exhibit strong antibacterial effects by generating reactive oxygen species (ROS), disrupting bacterial cell walls, and interfering with cellular functions. Their small size allows them to penetrate cells and damage DNA or proteins, leading to bacterial death. The antibacterial activity of the synthesized ZnO nanoparticles (ZnO NPs) was evaluated against both Gram-positive and Gram-negative bacterial pathogens, specifically *Staphylococcus aureus* (ATCC 25923) and *Pseudomonas aeruginosa* (ATCC 27853). The agar well diffusion method was employed to assess the antibacterial efficacy of the ZnO NPs. The results demonstrated a concentration-dependent antibacterial effect against the tested microorganisms. The ZOI was measured as 22 ± 0.20 mm for *S. aureus* and 20 ± 0.50 mm for *P. aeruginosa* (Fig. 6A, B). Interestingly, ZnO NPs exhibited greater efficiency against Gram-negative bacteria. This enhanced activity may be attributed to the porous structure of the Gram-negative cell wall, which facilitates the penetration of ZnO NPs, a feature not observed in the thicker peptidoglycan layer of Gram-positive bacteria. The results were compared with the earlier reports^44–47^ which confirms the significant antibacterial activity. Pioverdine (a siderophore produced by *P. aeruginosa*) mediated synthesized ZnO NPs show antibacterial activity against *P. aeruginosa* with ZOI of 20 mm^44^. In another study, ROS-mediated synthesized ZnO NPs showed activity against *P. aeruginosa* with ZOI of 16 mm^45^. Chemically synthesized ZnO NPs show ZOI of 22 mm against *P. aeruginosa* bacteria^47^ which is equal to our results. These results suggest our neem flower-mediated ZnO NPs show good to better antibacterial activity against bacterial pathogens and could serve as a potent antibacterial agent.

### Antifungal activity

Metal oxide NPs also show antifungal properties by inhibiting spore germination, disrupting fungal membranes, and producing oxidative stress. They can impair fungal growth and reproduction, making them effective against a broad range of fungal pathogens. The antifungal activity of zinc oxide nanoparticles (ZnO NPs) plays a crucial role in developing advanced therapeutic strategies for combating fungal infections, particularly in the face of increasing resistance to conventional antifungal agents. The unique properties of ZnO NPs enable them to target various fungal species, disrupt biofilm formation, and enhance the effectiveness of traditional treatments, making them an important tool in modern medicine. In this study, the antifungal potential of ZnO NPs was evaluated against *Candida tropicalis* (ATCC 10231) and *Candida glabrata* (MTCC 3019), with ketoconazole used as a control. The zone of inhibition (ZOI) observed was 23 ± 0.10 mm and 22 ± 0.20 mm at a concentration of 100 µg/mL (Fig. 6C, D; Table 1). These findings are consistent with previous reports, highlighting the promising potential of ZnO NPs as effective antifungal agents^48–50^. S. officinalis extract-mediated ZnO NPs show 22 and 15 mm of ZOI against *C. tropicalis* and *C. glabrata* which indicates that our ZnO NPs show better results^49^. Pomegranate peel extract-mediated ZnO NPs show ZOI of 23 and 18 mm which is also lower than our results^50^. It is confirmed that neem flower extract-mediated ZnO NPs serves as a better antifungal activity than earlier reports.

**Table 1:** ZOI of bacterial and fungal strains.

S. no	Strain name	(Zone of inhibition(mm)/concentration level (µg/ml)				
		Control	25	50	75	100
Antibacterial						
1.	*S. aureus*	24	10	14	18	22
2.	*P. aeruginosa*	24	8	13	17	20
Antifungal						
1.	*C. tropicalis*	27	19	20	22	23
2.	*C. glabrata*	27	18	19	21	22

### DNA binding activity

**Fig. 7:**
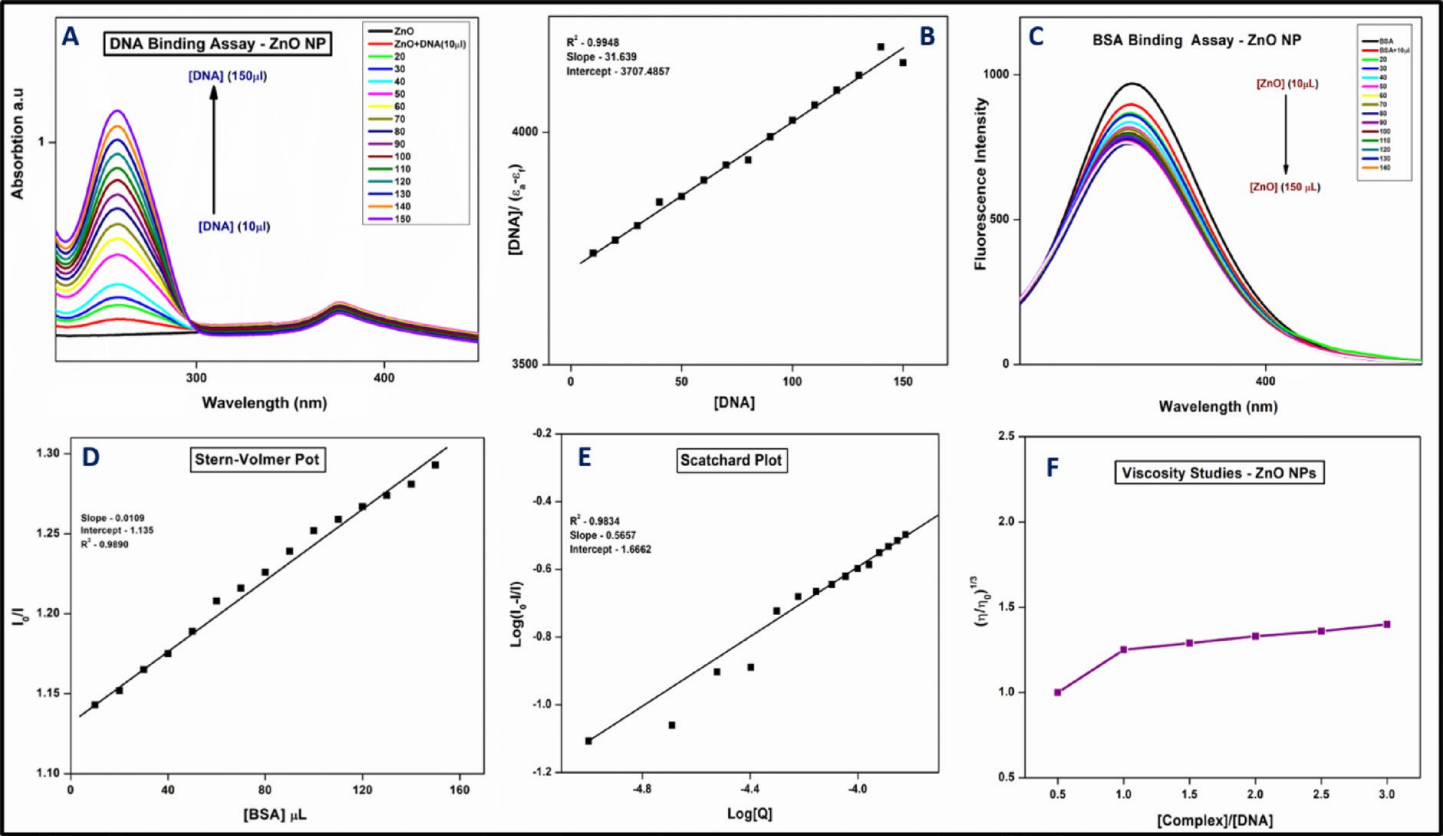
(**A**,**B**) DNA binding assay and linear plot. (**C**–**E**) BSA binding, Stern-Volmer plot, and Scatchard Plot (**F**) viscosity studies of ZnO NPs.

Metal oxide nanoparticles can interact with DNA through electrostatic forces, hydrogen bonding, or intercalation. These interactions may lead to conformational changes or DNA cleavage, useful in gene delivery or anticancer applications. The UV-visible titration method was employed to evaluate the binding efficacy of zinc oxide nanoparticles with calf thymus DNA, aiming to assess their potential for cancer cell targeting and DNA interaction. DNA, as a primary pharmacological target for anticancer therapies, plays a key role in determining the binding characteristics of these agents. The results revealed a hyperchromic shift in absorbance intensity with increasing DNA concentration, indicating both covalent and noncovalent binding modes. These binding interactions include electrostatic interactions and groove or intercalative binding. The intrinsic binding constant was calculated to be 8.539 × 10^5^ M^− 1^, suggesting a strong affinity between ZnO NPs and DNA exhibiting the base stacking interactions indicating groove binding (Fig. 7A, B)^26^. The present results were compared with earlier reports of green synthesized ZnO NPs from *Macrotyloma uniflorum* extract with CT-DNA. From the results, it is noted that the ZnO NPs shows a binding constant of 1.5 × 10^3^ M^− 1^ which is lower than our results. Our results shows a higher binding constant than earlier reports^51^. Molecules that bind to DNA can influence processes like replication, transcription, and repair, and may protect DNA from oxidative damage. Since ZnO NPs show good antioxidant activities and also show good binding properties with DNA results, they are potential candidates for therapeutic applications via protecting DNA in diseases linked to oxidative stress i.e., cancer, and neurodegenerative disorders^52–54^.

### BSA binding activity

Metal oxide NPs can bind to BSA, a model protein for studying protein-nanoparticle interactions. This binding affects the nanoparticles’ stability, bio-distribution, and cellular uptake, influencing their behavior in biological systems. The fluorescence titration method was employed to evaluate the binding efficacy of zinc oxide nanoparticles (ZnO NPs) with BSA, a structural analogue of HSA. In both static and dynamic modes, metal complexes bind to proteins, leading to quenching of the intrinsic fluorescence of aromatic residues such as phenylalanine, tyrosine, and tryptophan. In this study, BSA exhibited fluorescence at 345 nm, which showed a significant decrease as the concentration of ZnO NPs increased (Fig. 7C). The quenching constants (Kq) and binding constants (K_BSA_) were determined using the Stern-Volmer equation (v). The values for Kq and K_BSA_ were calculated to be 9.60 × 10^12^ M^− 1^ and 0.0960 × 10^6^ M^− 1^, respectively. The binding constant (K) was found to be 0.339 × 10^4^ M^− 1^. Furthermore, the number of binding sites and the binding constants were determined using the Scatchard equation (vi) (Fig. 7D, E). These results strongly suggest that ZnO NPs exhibit a high binding affinity to serum albumin, supporting their potential application in cancer treatment due to their robust binding nature.

### Viscosity studies

Viscosity measurement is an effective parameter for studying the binding of ZnO NPs to DNA, providing insights into the nature of their interaction. An increase in viscosity suggests intercalation, while minimal changes indicate electrostatic or groove binding, and a decrease in viscosity points to DNA condensation or aggregation. In our study, minimal or no change in viscosity was observed (Fig. 7F), suggesting that ZnO NPs bind strongly to DNA through groove binding. This observation aligns with previous findings from UV-Vis titration experiments.

###  In-vitro cytotoxicity

**Fig. 8:**
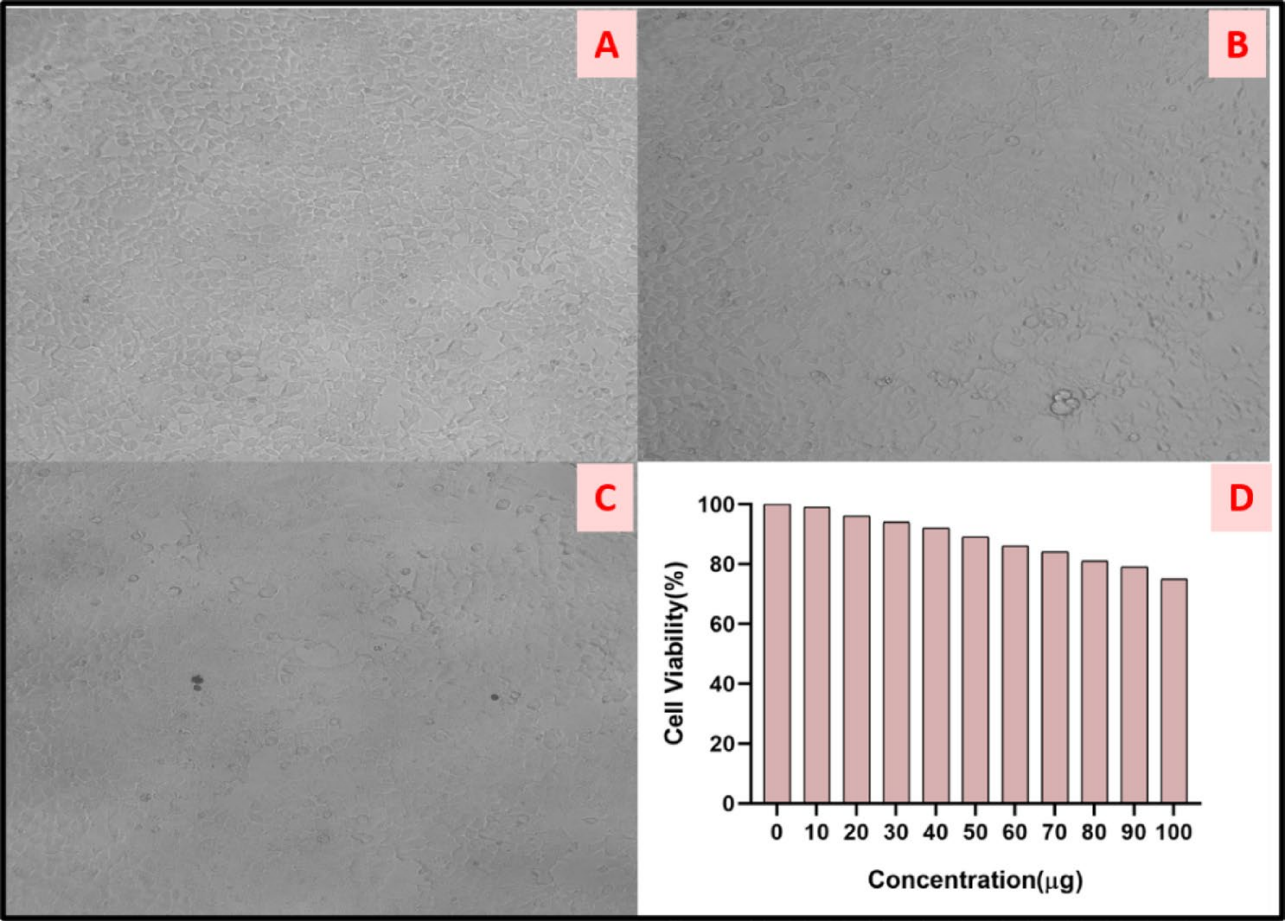
HEK-293 cells treated with ZnO NPs (**A**) control (**B**,**C**) treated cells (**D**) cell viability of ZnO NPs.

Metal oxide nanoparticles (MONPs) have shown promising antitumor activity due to their unique physicochemical properties, such as small size, high surface area, and ability to generate reactive oxygen species (ROS). This strong ROS generation induces apoptosis in breast and lung cancer cells. To evaluate the cytotoxicity and cell viability of the nanoparticle formulation, the MTT assay was conducted using cisplatin as the positive control and DMSO as the solvent control. Various concentrations of NF-ZnO NPs, ranging from 10 µg/mL to 100 µg/mL, were tested against normal HEK-294 cells (Fig. 8A–C). The results demonstrated that cell viability remained above 80% even at the highest concentration of 90 µg/mL, indicating that NF-ZnO NPs exhibit low toxicity within the tested concentration range (Fig. 8D). These findings are consistent with previous reports^55,56^ on the low toxicity of metal oxide nanoparticles in non-cancerous cell lines, further supporting their potential for safe clinical application. In comparison with the earlier reports, our ZnO NPs show a better % of viability than *S. Chirayita* leaf extract-mediated ZnO NPs^55^. The IC_50_ values of these NPs were found as < 100 µg/mL (neem) and 615 µg/mL (*S. chirayita*) respectively. These results indicate better cell viability of neem flower-based ZnO NPs on normal cells with less toxicity.

## Conclusion

This study explores the green synthesis of zinc oxide nanoparticles (ZnO NPs) using neem flower extract as both a reducing and stabilizing agent. The synthesized ZnO NPs were extensively analyzed with various analytical tools, confirming their successful production. Their biological potential was assessed through various in vitro assays, including antioxidant, antibacterial, and antifungal evaluations, which revealed significant inhibitory effects, highlighting their therapeutic potential. Furthermore, the binding interactions of ZnO NPs with DNA and bovine serum albumin (BSA) were analyzed, demonstrating strong binding affinities and suggesting their applicability in biomedical processes such as DNA modification. The cytotoxicity of ZnO NPs was also evaluated on HEK-293 cell lines, showing minimal toxicity to normal cells. Overall, the green-synthesized ZnO NPs demonstrate notable biological activities, such as strong antioxidant, antibacterial, and antifungal effects, as well as significant DNA and protein binding abilities. Their low cytotoxicity further highlights their potential for biomedical uses, especially in drug delivery and gene therapy. Further investigations can assess their potential in antimicrobial treatments, cancer therapy, and drug delivery, ensuring both effectiveness and safety for medical applications. In agriculture, they may serve as sustainable alternatives for pesticides, fertilizers, and soil conditioners, promoting eco-friendly farming. Their use in environmental purification, such as wastewater treatment and air filtration, requires further study to enhance their ability to eliminate contaminants and heavy metals. Additionally, their integration into energy storage systems, photocatalysts, and biosensors presents exciting prospects for green technology. Ongoing research on green-synthesized ZnO NPs can drive innovations that support environmental sustainability, public health, and technological advancement, making them highly valuable for future scientific and industrial developments.

## Electronic supplementary material

Below is the link to the electronic supplementary material.

Supplementary Material 1

## Data Availability

The data used in this study can be obtained upon request from the corresponding author.
