# Supplementary material for: Binding properties and biological applications of green synthesized ZnO nanoparticles from neem flower
Source: Sci Rep. 2025 May 22;15:17727. doi: 10.1038/s41598-025-02157-x (PMC12095587; doi:10.1038/s41598-025-02157-x)
Supplement: Supplementary file 1 — Supplementary Material 1 [file 41598_2025_2157_MOESM1_ESM.docx]

**Supporting Information**

**Binding Properties and Biological Applications of Green Synthesized ZnO Nanoparticles from Neem Flower**

Palanivelmurugan Mohanasundaram^1^, Mary Saral Antoneyraj^1*^

*^1^Department of Chemistry, School of Advanced Sciences, Vellore Institute of Technology, Vellore, Tamil Nadu, India – 632014.*

*Corresponding Author email-ID: [amarysaral@vit.ac.in](mailto:amarysaral@vit.ac.in), [marysaralvit@gmail.com](mailto:marysaralvit@gmail.com)

| **S.No** | **Content** | **Page No** |
| --- | --- | --- |
| 1 | General information of Characterisation techniques | 3 |
| 2 | Procedure for Cytotoxicity Assay | 3 |

**Characterisation:**

The synthesized ZnO nanoparticles (NPs) were characterized using a range of analytical techniques, including FTIR, UV-Vis spectroscopy, XRD, BET, PL, zeta potential analysis, TEM, and FE-SEM. FTIR analysis was conducted with a SHIMADZU IR Affinity-1 spectrometer over a wavelength range of 4000−1 to 400−1 cm⁻¹, using anhydrous KBr pellets for sample preparation. Elemental composition was determined using an Elementar Vario EL III instrument. Optical properties were examined with a JASCO V-670 UV-Vis spectrophotometer. X-ray diffraction (XRD) patterns were obtained using a BRUKER D8 Advance diffractometer, with crystalline size and structure estimated via the Scherrer equation (D = Kλ/βcosθ). Morphological and structural analyses were carried out through field emission scanning electron microscopy (FE-SEM) and energy-dispersive X-ray spectroscopy (EDX) using an FEI Quanta 250 FEG system, along with high-resolution transmission electron microscopy (TEM) using a JEOL JEM-2100 Plus. Zeta potential and dynamic light scattering (DLS) studies, performed with a Horiba SZ-100, assessed the stability of the ZnO NPs.

**Cytotoxicity**

Monolayer cell cultures were trypsinized, and the cell concentration was adjusted to 1.0 × 10⁵ cells/mL in a medium supplemented with 10% FBS. A 100 µL aliquot of this cell suspension (50,000 cells per well) was seeded into a 96-well microtiter plate. After 24 hours of incubation, allowing a partial monolayer to form, the supernatant was carefully removed, and the wells were gently rinsed with fresh medium. Subsequently, 100 µL of various concentrations of the test drugs were added to the wells. The plates were then incubated at 37°C with 5% CO₂ for 24 hours. Following this incubation, the test solutions were aspirated, and 100 µL of MTT solution (5 mg/10 mL in PBS) was added to each well. The plates were incubated for another 4 hours at 37°C in a 5% CO₂ environment. After incubation, the supernatant was removed, and 100 µL of DMSO was added to each well to dissolve the formazan crystals. The plates were gently shaken, and absorbance was measured at 590 nm using a microplate reader. The percentage of cell growth inhibition and the IC_50_ value were calculated from the dose-response curves.

**% Inhibition =** 100 – (OD of sample/OD of Control) x 100
